# Characterization of a *Mycobacterium smegmatis uvrA *mutant impaired in dormancy induced by hypoxia and low carbon concentration

**DOI:** 10.1186/1471-2180-11-231

**Published:** 2011-10-18

**Authors:** Angela Cordone, Bianca Audrain, Immacolata Calabrese, Daniel Euphrasie, Jean-Marc Reyrat

**Affiliations:** 1 Dipartimento di Biologia Strutturale e Funzionale, Università Federico II di Napoli, via Cinthia 4, 80126 Napoli, Italy; 2 Inserm, U570, Unité de Pathogénie des Infections Systémiques, Paris Cedex 15, 156 rue de Vaugirard, 75015 Paris, France; 3 Institut Pasteur, CNRS URA 2172, Unité de Génétique des Biofilms, 25-28 rue du Docteur Roux, 75724 Paris, France

## Abstract

**Background:**

The aerobic fast-growing *Mycobacterium smegmatis*, like its slow-growing pathogenic counterpart Mycobacterium tuberculosis, has the ability to adapt to microaerobiosis by shifting from growth to a non-proliferating or dormant state. The molecular mechanism of dormancy is not fully understood and various hypotheses have been formulated to explain it. In this work, we open new insight in the knowledge of *M. smegmatis *dormancy, by identifying and characterizing genes involved in this behavior.

**Results:**

In a library generated by transposon mutagenesis, we searched for *M. smegmatis *mutants unable to survive a coincident condition of hypoxia and low carbon content, two stress factors supposedly encountered in the host and inducing dormancy in tubercle bacilli. Two mutants were identified that mapped in the *uvrA *gene, coding for an essential component of the Nucleotide Excision Repair system (NER). The two mutants showed identical phenotypes, although the respective transposon insertions hit different regions of the *uvrA *gene. The restoration of the *uvrA *activity in *M. smegmatis *by complementation with the *uvrA *gene of *M. tuberculosis*, confirmed that i) *uvrA *inactivation was indeed responsible for the inability of *M. smegmatis *cells to enter or exit dormancy and, therefore, survive hypoxia and presence of low carbon and ii) showed that the respective *uvrA *genes of *M. tuberculosis *and *M. smegmatis *are true orthologs. The rate of survival of wild type, *uvrA *mutant and complemented strains under conditions of oxidative stress and UV irradiation was determined qualitatively and quantitatively.

**Conclusions:**

Taken together our results confirm that the mycobacterial NER system is involved in adaptation to various stress conditions and suggest that cells with a compromised DNA repair system have an impaired dormancy behavior.

## Background

*Mycobacterium tuberculosis*, the etiological agent of tuberculosis, has the ability to enter human macrophages and survive inside them in a 'latent' or 'non-proliferating' form for a long period of time. This behavior is termed dormancy or latency. During their lifetime, latent bacilli can reactivate giving rise to active tuberculosis, the transmissible form of the disease [1-3].

The molecular mechanism allowing dormancy is not fully understood due the lack of experimental systems that can closely mimic human latent infections [1]. In the granuloma, dormancy is hypothesized to occur in response to low oxygen, stress and lack of nutrients [1].

Experimental evidences suggest that, within the granuloma, the *in vivo *environment where dormant mycobacteria persist, the oxygen concentration is the limiting factor for bacterial growth and the condition that induces dormancy. Therefore, during the last few years, various experimental models using microaerobiosis or anaerobiosis, have been developed to reproduce dormancy *in vitro *[4-6]. There is also evidence that tubercle bacilli suffer nutrient deprivation in lung lesions [7]. Conditions of nutrient limitation have been used to investigate the ability of *M. tuberculosis *to persist in a non-growing state for long periods of time [7-9]. Importantly, dormancy is a common behavior to both pathogenic and non-pathogenic mycobacteria, *in vitro *[4,10,11], allowing the study of pathogenic species by using non-pathogens as model.

*M. smegmatis *is a fast growing non pathogenic mycobacterium frequently used as a model system to study its pathogenic counterpart *M. tuberculosis*. *M. smegmatis *becomes dormant in low oxygen concentration conditions [5] and remains viable for over 650 days when it suffers carbon, nitrogen and phosphorous-starvation [12]. Based on these observations, we decided to use low oxygen and limiting nutrient conditions to develop an *in vitro *system. Then, we used such system to screen a library of *M. smegmatis *generated by insertion mutagenesis and look for mutants defective in dormancy [13]. This strategy allowed the isolation of two mutants with insertions mapping in the *uvrA *gene. The UvrA protein belongs to the nucleotide excision repair system (NER) and is highly conserved among mycobacteria. NER counteracts the deleterious effects of DNA lesions acting as an endonuclease enzyme complex including four Uvr proteins: UvrA, UvrB, UvrC, and UvrD. UvrA, togheter with UvrB, plays a key role in the recognition of DNA damaged sites [14]. UvrC, together with UvrB, perform a single strand incision at both sides of the damaged site and the DNA fragment is removed by the action of the UvrD helicase.

While this DNA-repair system has been largely analyzed in *E. coli *[14], it remains poorly characterized in mycobacteria. It has been recently reported that the *M. smegmatis *genome is predicted to encode two additional UvrA proteins, named UvrA2 and UvrA-like protein, whose function are still unknown [15].

Here we report that the *M. smegmatis *UvrA protein is essential for the mycobacterial dormancy behavior and survival in hostile growth conditions, such as low oxygen and carbon content, also observed in the granuloma. Our results, together with recent analyses [16-19], suggest that the NER system plays a key role in *M. smegmatis *dormancy.

## Results

### *M. smegmatis *dormancy is induced under conditions of low oxygen and low carbon availability

**Figure 1:**
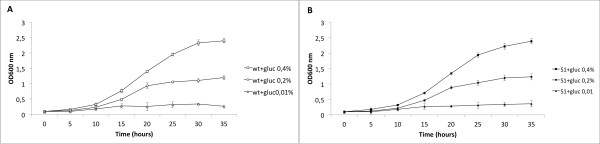
**Effect of nutrient limitation on *M. smegmatis *growth**. (A) *M. smegmatis *wild type and (B) S1 strains were grown in M9 minimal medium supplemented with glucose at the final concentration of 0.4% (wt, white square; S1, black square); 0.2% (wt, white circle; S1, black circle) or 0.01% (wt, white triangle; S1, black triangle). The growth rate was monitored for 35 hours by measuring OD_600nm_. For each strain the data reported in graph represent the mean of three independent experiments.

**Figure 2:**
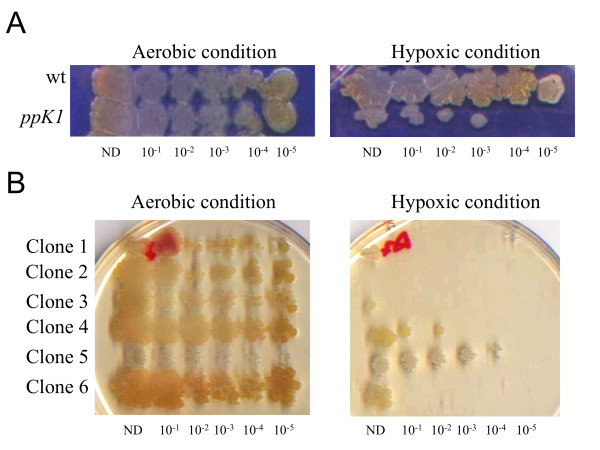
**Screening of *M. smegmatis *mutant library**. A) (Left panel) *M. smegmatis *wild type and *ppk *mutant were grown in M9 minimal medium supplemented with glucose 0.2% until OD_600nm _= 1.0, serially diluted up to 10^-5 ^and transferred by using a metal replicator on agar plates. (Right panel) After incubation at 37°C for 4-5 days for aerobic cultures, or incubation for 2 weeks in an AnaeroGen gas pack system at 37°C followed by incubation under aerobic condition at 37°C for 4-5 days, plates were compared. B) Individual screening of 6 selected mutants. Each clone was grown in M9 minimal medium supplemented with glucose 0,2% until OD_600nm _= 1.0, serially diluted up to 10^-5 ^and transferred by using a metal replicator on agar plates. Clones 1, 3 and 6 were considered as moderately affected clones. Clone 2 was considered as severely affected. ND = Non Diluted culture

In order to develop a simple and reliable strategy to screen a *M. smegmatis *library for mutants unable to grow in conditions of hypoxia and low carbon concentration, we first compared the effects of these conditions on the dormancy behavior of *M. smegmatis *wt and *ppk1*- mutant cells [the latter were used as a control as they have been recently reported to be sensitive to hypoxic condition [20]]. To assess whether low carbon availability represented a limiting factor, we analyzed the effect of different glucose concentrations on *M. smegmatis *growth rate. To this purpose, wt and *ppk1 *strains were grown at 37°C in minimal medium containing glucose as the only carbon source at the following final concentrations: 0.4%; 0.2% or 0.01% (w/v). The growth rate was monitored for 35 hours by measuring the OD_600nm_. As shown in Figure 1A, when the minimal medium was supplemented with glucose 0.4% (w/v), cultures entered stationary phase at an OD_600nm _of 2.4, whereas using glucose 0.2% (w/v), stationary phase was entered at 1.1 OD. When an even lower glucose concentration (0.01% w/v) was added to the medium, cells growth was inhibited, indicating that the arrest of cell growth was due to carbon starvation. Similar results were obtained for the *ppk *mutant (data not shown). These results indicate that the *M. smegmatis *growth rate is significantly limited by the amount of carbon source. Based on this, we decided to use a glucose concentration of 0.2% for the further analyses. Next, we analyzed the effect of hypoxia on dormancy by following the bacterial cell growth up to 1.0 OD in the presence of 0.2% gluscose. Serial dilutions of wt and *ppk1*- strains were transferred to agar plates and incubated in either atmosphere oxygen concentration or anaerobic conditions in jar (< 1%O_2_). Bacterial cell growth of both wt and *ppk1 *strains, resulted unaffected in aerobic conditions, for as long as 4-5 days of incubation. However, the cell growth of the two strains resulted completely inhibited in anaerobic conditions for at least 14 days, indicating that low oxygen is an inhibitory factor. After 14 days of growth in anaerobic conditions, the same plates containing wt and *ppk1 *cells were incubated in normal oxygen condition for 4-5 day. As represented in Figure 2A, *M. smegmatis *wild type cells show restored cell growth without a significant cell loss, when exposed to oxygen. This result indicates that wt cells are able to exit the dormant state and restore cell growth. In contrast, *ppk-1 *cells showed only a 40% of restored cell growth in compared to wt (data not shown), suggesting that this strain is unable to either enter or exit the dormant state. These results allow us to conclude that our experimental system represents a valuable platform to screen the *M. smegmatis *transposon library.

### Library screening and isolation of *M. smegmatis *mutants with impaired dormancy behavior upon hypoxia and low carbon availability

Ten thousand clones of a transposon library containing more than 20,000 mutants and covering the majority of the *M. smegmatis *gene pool [13] were screened as described above to isolate mutants unable to survive a prolonged exposure to low oxygen tension and low carbon availability. The screening allowed us to isolate a total of 278 insertion mutants unable to survive these conditions. Each clone was serially diluted to further confirm the observed phenotype (see a 6-clone sample plate in Figure 2B). During individual screening, 21 clones sensitive to hypoxia and low carbon availability were isolated and divided in two groups: the first group included 8 clones that were completely unable to survive and, therefore, defined as severely affected (S); the second group included the remaining 13 clones that were only partially affected and, therefore, defined as moderately affected (M) (Figure 2B). Most likely, these mutants are unable to either enter or exit the dormant state.

In order to identify the sites of transposon insertions, the genomic DNA of all clones was extracted, digested with the *Sal*I restriction enzyme and used as template in Ligated Mediated (LM)-PCR reactions [21]. Using this approach, we were able to map the site of transposon insertion of 13 M mutants and 3 S mutants (Table 1). In two independent mutants, here named S1 and S2, the transposon insertion mapped in different positions of the *uvrA *gene (Table 1). The *uvrA *gene encodes the UvrA protein that belongs to the nucleotide excision repair system (NER). As the two mutants showed identical phenotypes, S1 was chosen for further characterization.

**Table 1:** Genes disrupted in M and S mutants identified ( LM)-PCR

Clone name^3 ^	*M. smegmatis *mc2155^b^	Gene product/function	Insertion site^c^	*M. Tuberculosis *ortholog(% identity)*^d^
Ml	MSMEG_4806	putative acyl-CoA	201	NF^e^
M2	MSMEG6781	hypothetical protein	681	NF
M3	MSMEG_4727	Mycocerosic Acid synthase	2723	Rv1527(65%)
M4	MSMEG_6215	Manganese containing catalase	252	NF
M5	MSMEG_5925	Riesce(2Fe-2S) domain protein	608	Rv3526 (65%)
M6	MSMEG_3215	ABC transporter ATP-binding protein	518	NF
M7	MSMEG_5714	Short-chain dehydrogenase/reductase SDR	104	NF
M8	MSMEG_0200	Hypotetical protein	779	NF
M9	MSMEG_6105	Cell division protein	447	Rv3610c (86%)
M10	MSMEG_0831	Short-chain dehydrogenase	159	NF
Mil	MSMEG_6611	Hypotetical protein	110	NF
M12	MSMEG_0304	Acyl-CoA synthase	177	Rv1427c (77%)
M13	MSMEG_0228	Adenylate and Guanilate cyclase domain protein	1417	NF
SI	MSMEG_3808	UvrA exinuclease, ABC, A subunit	866	Rv1638 (88%)
S2	MSMEG_3808	UvrA exinuclease, ABC, A subunit	959	Rv1638 (88%)
S3	MSMEG_4293	Glutammate-ammonia-ligase adenylyltransferase	883	Rv2221c(73%)

^a^Selected mutants moderately (M) or severely affected (S)

^b^Genes are indicated as annotated by TIGR/GeneBank database (Accession number C000480.1)

^c^Relative to the first base of the putative coding sequence

^d^Cut off identity was set at 60%

^e ^Not found

### UvrA is important for mycobacterial dormancy and survival upon hypoxia

**Figure 3:**
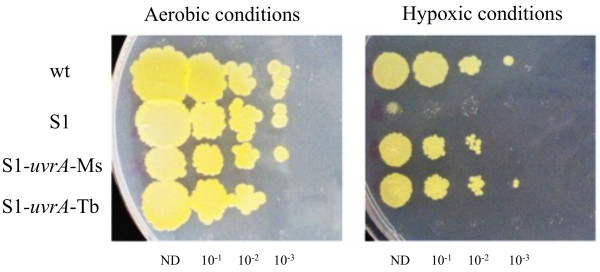
**Effect of hypoxia and low carbon content on *M. smegmatis *dormancy**. *M. smegmatis *wild type, S1 (*uvrA*::tn611), S1-*uvrA*-Ms and S1-*uvrA*-Tb strains were grown in M9 minimal medium supplemented with glucose 0.2% until OD_600nm _= 1.0. Bacterial cultures were then serially diluted up to 10^-5 ^and transferred to agar plates. After incubation at 37°C for 4-5 days for aerobic cultures, or 2 weeks for anaerobic cultures in an AnaeroGen gas pack system at 37°C followed by incubation under aerobic condition at 37°C for 4-5 days, plates were compared. ND = Non Diluted culture

To verify whether the severe dormancy defect of the *uvrA *mutants in our *in vitro *model system was a direct effect of UvrA deficiency, we performed complementation analyses. A wild type allele of the *uvrA *gene was PCR-amplified, cloned into the integrative expression vector pNip40-b [22] and electroporated into the S1 mutant strain. The resulting strain was analyzed for its phenotype. As shown in Figure 3, the reintroduction of a single copy of *uvrA *from *M. smegmatis *(here defined as S1-*uvrA*-Ms) fully restored the dormancy defect of the parental mutated strain. Identical results were obtained for the S2 mutant (data not shown).

As shown in Table 1, a BLAST search performed using *uvrA *of *M. smegmatis *as a query showed that this gene is highly conserved in *M. tuberculosis*. The orthology between the *M. smegmatis *and *M. tuberculosis *UvrA proteins was verified by using the *M. tuberculosis uvrA *gene to complement the *M. smegmatis uvrA *deficient strain (Figure 3). The reintroduction of the *M. tuberculosis uvrA *wt gene (here defined as S1-*uvrA*-Tb) was able to restore the wt phenotype in the *M. smegmatis *mutated strain. Our results demonstrate that UvrA is essential for *M. smegmatis *to enter or exit dormancy upon hypoxia. Moreover, we proved that the *M. smegmatis *and *M. tuberculosis *gene products are true orthologs.

### UvrA deficiency does not influence *M. smegmatis *growth under nutrient limiting conditions

In addition to hypoxia, nutrient starvation is also supposed to affect cell growth. To check whether the NER deficiency had an effect on cell growth in nutrient limitation, we monitored the growth rate of the *uvrA *mutant and the complemented strains in minimal medium supplemented with the following final glucose concentration: 0.4%; 0.2% or 0.01% (w/v). As shown in Figure 1B, *uvrA *mutant cells grown in 0.2% glucose entered stationary phase at a lower optical density (OD_600nm_≈1.1) in compared to cells of the same strains grown in higher (0.4%) glucose concentration. Moreover, both wt and *uvrA *cell growth arrested at the limiting glucose concentrations (0.01%). Taken together these results indicate that *M. smegmatis *growth rate is limited by the amount of carbon available and also that absence of UvrA does not affect *M. smegmatis *growth under nutrient-limited conditions.

### The mycobacterial NER system is involved in the protection from UV-induced damage of DNA

**Figure 4:**
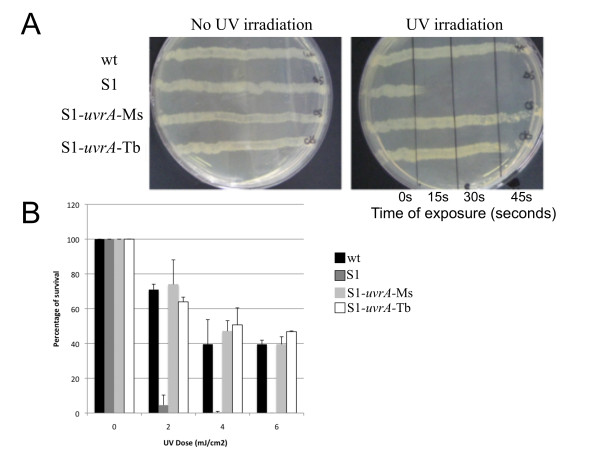
**UV irradiation assay**. A) *M. smegmatis *wild type, S1 (*uvrA*::tn611), S1-*uvrA*-Ms and S1-*uvrA*-Tb strains were streaked from left to right on LB plates. Plates were either exposed or not to UV radiation (0, 15, 30 and 45 seconds). B) *M. smegmatis *wild type, S1, S1-*uvrA*-Ms and S1- *uvrA*-Tb cells in exponential phase were harvested and resuspended in PBS (see Methods for details). Aliquots were exposed to different UV doses (0, 2, 4 and 6 mJ/cm^2^). The percentage of survival of each strain was determined and represented as the mean value of three independent experiments.

The NER system has been extensively studied in *E. coli *where the *uvr *gene products protect bacteria from different types of DNA damages including those induced by UV radiations [14]. To verify whether the NER system had a similar function in mycobacteria, we measured the effect of UV light exposure on wild type, *uvrA *(S1), the complemented derivatives of this mutant, containing the *uvrA *gene from *M. smegmatis *(S1-*uvrA*-Ms) and *M. tubercolosis *(S1-*uvrA*-Tb), respectively. As shown in Figure 4A, while *uvrA *cells were unable to grow after a 15 sec exposure to UV light (λ = 254 nm), the wild type and the complemented strains were unaffected by the treatment. To further verify the importance of UvrA in preventing UV-induced DNA damages, all strains were exposed to different UV light doses. As shown in Figure 4B, the S1 strain showed a marked sensitivity to UV irradiation with only 7% survival after exposure to 2 mJ/cm^2 ^UV, whereas the wild type and both complemented strains showed a comparable dose-dependent sensitivity to UV irradiation with more than 60% survival after exposure to the same UV dose. Taken together these results suggest that *M. smegmatis *UvrA is involved in the repairing of UV-induced DNA damages as reported for other bacteria [14].

### The UvrA NER system contributes to repair DNA oxidative damages

It is hypothesized that inside the granuloma, dormant bacilli are continuously exposed to reactive oxygen species (ROS) and Reactive Nitrogen Intermediates (RNI) [23-27], lipo-soluble molecules that can enter the mycobacterial waxy cell wall, thus causing DNA damages.

**Figure 5:**
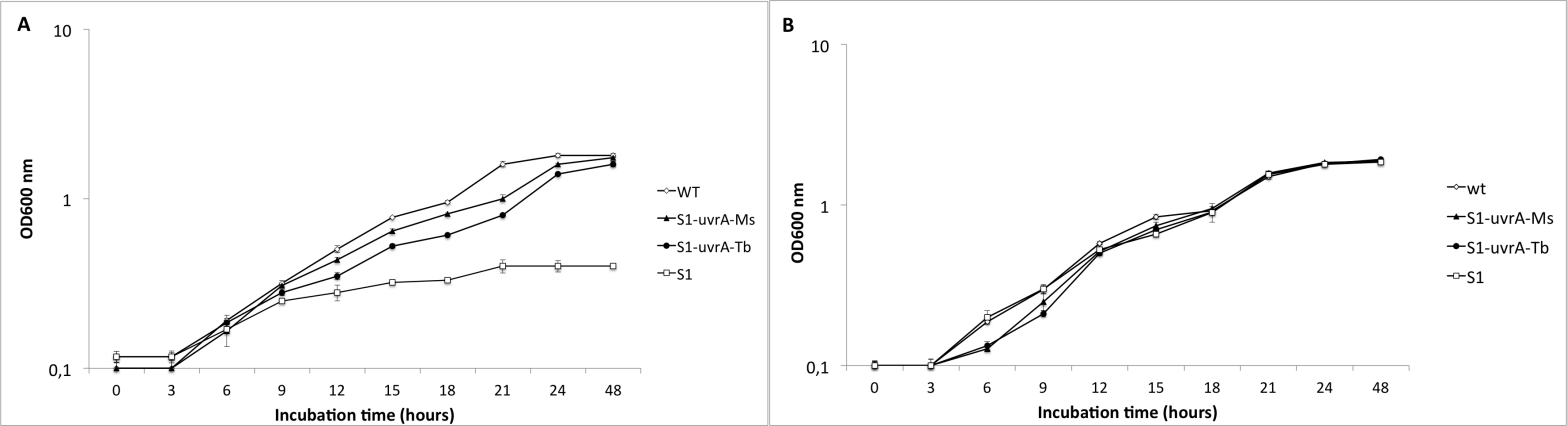
**Effect of hydrogen peroxide on cell growth**. *M. smegmatis *cells of wild type, S1, S1-*uvrA*-Ms and S1- *uvrA*-Tb strains were grown in LBT with (A) or without (B) 5 mM H_2_O_2 _and OD_600nm _determined every 3 hours. For each strain the data reported in graph is the mean of three independent experiments.

To better clarify the role of the NER system in oxidative stress, we determined the effect of oxidative stress on wt, *uvrA *mutant S1 and complemented strains S1-*uvrA*-Ms and S1-*uvrA*-Tb cell growth. To this purpose, cells were incubated in the presence of 5 mM H_2_O_2 _and growth (OD_600nm_) was monitored at 3 hours intervals for 48 h. As shown in Figure 5A, the *uvrA *mutant strain, in contrast to wild type and complemented strains, stopped growing after three mass doubling time in the presence of hydrogen peroxide. The *uvrA *mutant strain reached a maximal cell density of 8 × 10^6 ^c.f.u. ml^-1^, which was approximately 4-fold higher than the density of the initial inoculum (2 × 10^6 ^c.f.u. ml^-1^) but 1000-fold less than the density of the wild-type and the two complemented strains (8 × 10^9 ^c.f.u. ml^-1^). Interestingly, the growth curve of the two complemented strains shows a lag-phase under normal growth conditions (Figure 5B) that it is not observed when bacteria are exposed to oxidative stress (Figure 5A). This result is probably due to the fact that, in the complemented strains, the *uvrA *gene is not expressed under the regulation of endogenous promoter region. Our results suggest that mycobacteria need a functional NER system to neutralize the damaging effects of oxyradicals, emphasizing once again the importance of the NER system for mycobacterial survival under stress conditions.

## Discussions

*In silico *analysis of mycobacterial genomes [28] has shown the presence of genes encoding enzymes involved in different DNA repair system such as Nucleotide Excision Repair (NER), Base Excition Repair (BER), Recombinational Repair, Non-Homologous End-joining repair and SOS repair. Surprisingly, even if mycobacteria lack the *mutSL*-based post-replicative mismatch repair system [29], their mutation rate is similar to those of other bacteria [30]. A recent analysis provided evidence that the mycobacterial NER system is able to repair a wider range of DNA damages than the corresponding *E. coli *system, highlighting its involvement in mismatch recognition and suggesting a crucial role of the NER system in preserving the mycobacterial genome integrity [16,19]. Although mycobacterial DNA repair systems are still not well characterized [31], it is possible that their functions are important for survival of tubercle bacilli during latency. Latent mycobacteria, in fact, are continuously exposed to the action of compounds such as Reactive Oxygen Species (ROS) and Reactive Nitrogen Intermediates (RNI) that induce DNA damage [24-27]. The deleterious effects of these intermediates, is probably counteracted by the synergic action of highly efficient and functional DNA repair systems. Oxidative stress results in different types of non-bulky DNA damages such as formation of abasic sites, single and double-stranded breaks, or production of oxidized bases converting guanine to 7,8-dihydro-8-oxoguanine. Although Base Excision Repair (BER) is the main pathway for the removal of this kind of lesion [32-34], we hypothesized that during dormancy the BER system is overwhelmed by extensive DNA damages and that mycobacterial genome integrity might be preserved by a synergic action of different DNA repair systems among which NER. Earlier studies have shown that a *M. tuberculosis *NER-deficient strain mutated in *uvrB*, is markedly attenuated for survival in mice and that UvrB protein is required for resistance of *M. tuberculosis *to both ROS and RNI species *in vivo *[17]. It has also been recently reported that a *M. smegmatis uvrB *mutant is sensitive to stress factors such as hypoxia, a condition under which bacteria are not proliferating thus they can accumulate DNA damage over time [18].

In this study we used hypoxia and low carbon availability as a model for dormant state to screen a library of *M. smegmatis *insertional mutants. This strategy led to the isolation of two strains mutated in the *uvrA *gene and unable to survive such condition. We showed that the *M. smegmatis *UvrA protein is essential to survive the *in vitro *dormancy condition of growth. Moreover, we demonstrated that the UvrA protein is needed for cell to neutralize both UV light- and oxyradicals-induced damages.

According to these data, it is possible to hypothesize that the *uvrA *mutant is not able to survive the *in vitro *dormancy conditions because of sudden oxygen increase following the opening of the jars. The oxidative burst created is probably neutralized by the synergic action of functional DNA repair systems, which maintain the genome integrity. A deficiency in one of the DNA repair systems during this step may result in the accumulation inside the mycobacterial genome of mutations which are not counteracted by the action of the remaining repair systems, resulting in failure of cells to reactivate.

A future analysis of the *M. tuberculosis uvrA *knock-out mutants using human macrophages and mouse infection as an *in vitro *and *in vivo *dormancy model systems will give more insight into mycobacterial survival during latency and will help to better clarify the importance of *M. tuberculosis *NER system during latency.

## Conclusions

In this report we describe the isolation and subsequent analysis of a *M. smegmatis *strain mutated in the *uvrA *gene under different stress conditions.

We demonstrate that *M. smegmatis *UvrA deficient strain is more sensitive to hypoxia, UV radiation and oxidative stress than wild type and that the use of *M. smegmatis *own gene or the corresponding *M. tuberculosis *homologous gene, fully restore the wild type ability to resist these factors.

Based on our data, we can conclude that UvrA protein, and thus the NER system, is an importatnt player for adaptation of *M. smegmatis *to various stress conditions. Further analysis are needed to better clarify the role of NER system in the complex phenomenon of mycobacterial dormancy.

## Methods

### Bacterial strains, media and growth conditions

*Mycobacterium smegmatis *mc^2^155 [35] is the parental of all the recombinant strains described below. *E. coli *DH5α strain (*supE44 ΔlacU169 [φ80ΔlacZM15] hsdR17recA1*) [36] was used for all cloning experiments.

*M. smegmatis *mc^2^155 and derivatives were grown in LB medium containing 0,05% Tween 80 (LBT).

For nutrient limitation experiments, *M. smegmatis *mc^2^155 and derivatives were grown in M9 containing 1 mM Mg_2_SO_4 _and supplemented with glucose at the following final concentrations: 0.4%; 0.2% or 0.01% (w/v).

*Escherichia coli *strains were grown in LB medium. When required, antibiotics were added to the medium at the following final concentrations: ampicillin 100 μg/ml, kanamycin 25 μg/ml. Hygromicin was used at 200 μg/ml for *E. coli *and 50 μg/ml for *M. smegmatis*.

### *In vitro *dormancy assay

*M. smegmatis *transposon insertion mutants [13] were thawed and printed by using a metal replicator in 96 well plates in M9 medium containing 1 mM Mg_2_SO_4 _and 0.2% glucose at 37°C in standing condition until OD_600_nm = 1.0. After incubation time, wild type and mutant strains were serially diluted 1:10 up to 10^-5 ^and spotted on M9 agar plates containing glucose. Control plates were incubated in normal atmosphere (20% O_2_) for 4-5 days at 37°C, whereas experimental plates were transferred to anoxic jar (Oxoid) for 2 weeks at 37°C. Hypoxia was generated using AnaeroGen gas pack system (Oxoid) inside jars and anaerobiosis (O2 <1%) was checked by using methylene blue as indicator. Plates were finally removed from the anoxic jar and incubated in normal atmosphere to enable growth of the surviving bacteria.

### DNA manipulation

Plasmid and chromosomal DNA preparation, restriction digestion, ligation, bacterial transformation and agarose gel electrophoresis were performed as described [36,37]. For complementation analyses, *uvrA *genes from *M. smegmatis *mc2 155 and *M. tuberculosis *H37Rv were PCR amplified as follow: the wild-type *uvrA *gene from *M. smegmatis *mc^2^155 was amplified by PCR with Pfu Turbo high fidelity DNA polymerase (Stratagene) by using chromosomal DNA as a template and oligos uvrA-Ms-F and uvrA-Ms-R (Table 2) as primers. Both primers contain an engineered XbaI restriction site. After purification with the PCR purification Qiagen kit, PCR products were digested with XbaI and cloned into the dephosphorylated integrative expression vector pNIP40b [22] at the unique XbaI site to generate pNIP-uvrA-Ms. As previously reported, cloning a gene at this site in pNip40b leads to a transcriptional fusion with an upstream promoter and expression of the transgene [38,39]. One clone was selected and sequenced. Plasmid pNip-uvrA-Tb was obtained using a similar strategy. Chromosomal DNA of *M. tuberculosis *H37Rv was amplified using primers uvrA-Tb-F and uvrA-Tb-R and Pfu Turbo high fidelity DNA polymerase (Stratagene). PCR product was purified with the PCR purification Qiagen kit, digested with XbaI and ligated into the pNIP40b at the unique XbaI site. One clone was selected and sequenced. These plasmids were electroporated into the *M. smegmatis uvrA *mutant strain S1 (*uvrA *::*Tn*611) and transformants were selected on hygromicin containing LB plates and named S1-*uvrA*-Ms and S1-*uvrA*-Tb.

**Table 2:** Synthetic oligonucleotides

Name	Sequence (5' - 3')^a^	Position of annealing ^b^
*uvrA-Ms-Y*	ctag**tctaga**gacgtgtccggtgtaggtgt	-180/-160
uvrA-Ms-R	ctag**tctaga**atgacctggtggatcgactg	+150/+169
uvrA-Tb-F	ctag**tctaga**cgatgccttgaggatcgtg	-258/-240
uvrA-Tb-R	ctag**tctaga**gaagatcgaaacccgatacg	+194/+213

^a ^Underlined is an unpaired tail carrying *Xbal *restriction site. ^b ^Position of annealing refers to the *uvrA *gene sequence, with the first base of the translational initiation codon as +1.

### Ligation-mediated PCR (LM-PCR)

Transposon insertions were mapped by using LM-PCR as previously reported [21]. LM-PCR reactions were done using SalI and BamHI enzymes (Roche). PCR products were separated by 1.5% agarose gel and the fragments were purified using QIAquick gel extraction kit (Qiagen). The purified fragments were used as templates in sequencing reactions together with oligonucleotide F or G [20].

### UV irradiation assay

*M. smegmatis *strains were grown in LBT medium up to exponential phase (OD_600nm _= 0.4-0.6). Samples from these cultures were streaked on LB agar plates. Plates were exposed to UV light during 0, 15, 30 and 45 seconds and then incubated at 37°C for 3-4 days. The percentage of survival of these strains after UV irradiation was also determined; exponential phase cultures of all strains were harvested and pellets were re-suspended in 2 mL of 1× PBS. 200 μL were exposed to UV intensities of 0, 2, 4 and 6 mJ/cm^2 ^(as measured with a VLX 3W dosimeter). Viable counts of the cultures were determined by plating serial dilution on LB plates with appropriate antibiotics after 4 days at 37°C.

### Hydrogen peroxide assay

*M. smegmatis *strains, were grown in triplicate in LBT medium up to stationary phase (OD_600 _= 1.5). Cultures were serially diluted 1:100 in LBT supplemented with 0 and 5 mM H_2_O_2 _freshly prepared, placed in the microtiter well plates and incubated in a Bioscreen C kinetic growth reader at 37°C with constant shaking. Growth was monitored as OD_600nm _at 3 h intervals for 48 h.

## Competing interests

The authors declare that they have no competing interests.

## Authors' contributions

AC carried out most of the experiments, contributed to experimental design and draft the manuscript; BA carried out complementation experiments and UV assay; IC carried out oxidative stress experiment; DE carried out LM-PCR experiment; JMR conceived and supervised the study. All authors read and approved the final manuscript.

## References

[B1] Manabe YC Bishai WR Latent Mycobacterium tuberculosis-persistence, patience, and winning by waiting Nat Med 2000 1327 1329 10.1038/82139 11100115

[B2] Gomez JE McKinney JD *M. tuberculosis *persistence, latency, and drug tolerance Tuberculosis 2004 84 29 44 10.1016/j.tube.2003.08.003 14670344

[B3] Honer zu Bentrup K Russel DG Mycobacterial persistence: adaptation to a changing environment TRENDS in Microbiology 2001 10.1016/s0966-842x(01)02238-7 11728873

[B4] Dick T Lee BH Murugasu-oei B Oxygen depletion induced dormancy in *Mycobacterium smegmatis* FEMS Microbiol Letters 1998 162 159 164 10.1111/j.1574-6968.1998.tb13040.x 9673018

[B5] Lim A Dick T Plate-based dormancy culture system for *Mycobacterium smegmatis *and isolation of metronidazole-resistant mutants FEMS Microbiol Letters 2001 200 215 219 10.1111/j.1574-6968.2001.tb10718.x 11425478

[B6] Wayne LG Hayes LG An in vitro model for sequential study of shiftdown of *Mycobacterium tuberculosis *through two stages of non replicating persistence Infect Immun 1996 64 2062 2069 8675308 10.1128/iai.64.6.2062-2069.1996 PMC174037

[B7] Nyka W Studies on the effect of starvation on mycobacteria Infect Immun 1974 9 843 850 4132910 10.1128/iai.9.5.843-850.1974 PMC414896

[B8] Loebel RO Shorr E Richardson HB The influence of foodstuffs upon the respiratory metabolism and growth of human tubercle bacilli J Bacteriol 1933 26 139 166 16559649 10.1128/jb.26.2.139-166.1933 PMC533551

[B9] Loebel RO Shorr E Richardson HB The influence of adverse conditions upon the respiratory metabolism and growth of human tubercle bacilli J Bacteriol 1933 26 167 200 16559650 10.1128/jb.26.2.167-200.1933 PMC533552

[B10] Lim A Eleuterio M Hutter B Murugasu-Oei B Dick T Oxygen depletion induced dormancy in *Mycobacterium Bovis *BCG J Bacteriol 1999 181 2252 2256 10094705 10.1128/jb.181.7.2252-2256.1999 PMC93640

[B11] Rustad TR Sherrid AM Minch KJ Sherman DR Hypoxia: a window into *Mycobacterium tuberculosis *latency Cell Microbiol 2009 11 1151 1159 10.1111/j.1462-5822.2009.01325.x 19388905

[B12] Smeulders MJ Keer J Speight RA Williams HD Adaptation of *Mycobacterium smegmatis *to stationary phase J Bacteriol 1999 181 270 283 9864340 10.1128/jb.181.1.270-283.1999 PMC103559

[B13] Sonden B Kocincova D Deshayes C Euphrasie D Rayat L Laval F Frahel C Daffè M Etienne G Reyrat JM Gap, a mycobacterial specific integral membrane protein, is required for glycolipid transport to the cell surface Mol Microbiol 2005 58 426 440 10.1111/j.1365-2958.2005.04847.x 16194230

[B14] Van Houten B Croteau DL DellaVecchia MJ Wang H Kisker C "Close-fitting sleeves": DNA damage recognition by the UvrABC nuclease system Mutat Res 2005 577 92 117 10.1016/j.mrfmmm.2005.03.013 15927210

[B15] Kurthkoti K Varshney U Base exision and nucleotide exision repair pathways in mycobacteria in press 10.1016/j.tube.2011.06.005 21764637

[B16] Darwin KH Nathan CF Role for nucleotide excision repair in virulence of *Mycobacterium tuberculosis* Infect Immun 2005 73 4581 458 10.1128/IAI.73.8.4581-4587.2005 16040969 PMC1201236

[B17] Darwin KH Nathan CF Role for nucleotide excision repair in virulence of *Mycobacterium tuberculosis* Infect Immun 2005 73 4581 458 10.1128/IAI.73.8.4581-4587.2005 16040969 PMC1201236

[B18] Kurthkoti K Kumar P Jain R Varshney U Important role of the nucleotide excision repair pathway in *Mycobacterium smegmatis *in conferring protection against commonly encountered DNA-damaging agents Microbiology 2008 154 2776 2785 10.1099/mic.0.2008/019638-0 18757811

[B19] Guthlein C Wanner RM Sander P Davis EO Bosshard M Jiricny J Bottger EC Springer B Characterisation of the mycobacterial NER system reveals novel functions of uvrD1 helicase J Bacteriol 2009 191 555 562 10.1128/JB.00216-08 19011038 PMC2620815

[B20] Sureka K Dey S Singh AK Dasgupta A Rodrigue S Basu J Kundu M Polyphosphate kinase is involved in stress-induced *mprAB-sigE-rel *signalling in mycobacteria Mol Microbiol 2007 65 261 276 10.1111/j.1365-2958.2007.05814.x 17630969

[B21] Prod'hom G Guilhot C Gutierrez MC Varnerot A Gicquel B Vincen V Rapid discrimination of *Mycobacterium tuberculosis *complex strains by ligation-mediated PCR fingerprint analysis J Clin Microbiol 1997 35 3331 3334 9399550 10.1128/jcm.35.12.3331-3334.1997 PMC230178

[B22] Berthet FX Lagranderie M Gounon P Laurent-Winter C Ensergueix D Chavarot P Thouron F Maranghi E Pelicic V Portnoï D Marchal G Gicquel B Attenuation of virulence by disruption of *Mycobacterium tuberculosis erp *gene Science 1998 282 759 762 9784137 10.1126/science.282.5389.759

[B23] Adams LB Dinauer MC Morgenstern DE Krahenbuhl JL Comparison of the roles of reactive oxygen and nitrogen intermediates in the host response to *Mycobacterium tuberculosis *using transgenic mice Tubercle Lung Dis 1997 78 237 246 10.1016/S0962-8479(97)90004-6 10209678

[B24] Akaki T Tomioka H Shimizu T Dekio S Sato K Comparative roles of free fatty acids with reactive nitrogenintermediates and reactive oxygen intermediates in expression of the anti-microbial activity of macrophages against *Mycobacterium tuberculosis* Clin Exp Immunol 2000 121 302 310 10.1046/j.1365-2249.2000.01298.x 10931146 PMC1905686

[B25] Nathan C Shiloh MU Reactive oxygen and nitrogen intermediates in the relationship between mammalian hosts and microbial pathogens P Natl Acad Sci USA 2000 97 8841 8848 10.1073/pnas.97.16.8841 PMC34021 10922044

[B26] Lau YL Chan GC Ha SY Hui YF Yuen KY The role of the phagocytic respiratory burst in host defense against *Mycobacterium tuberculosis* Clin Infect Dis 1998 26 226 227 10.1086/517036 9455564

[B27] Wang CH Liu CY Lin HC Yu CT Chung KF Kuo HP Increased exhaled nitric oxide in active pulmonary tuberculosis due to inducible NO synthase upregulation in alveolar macrophages Eur Respir J 1998 11 809 815 10.1183/09031936.98.11040809 9623681

[B28] Mizrahi V Andersen SJ DNA repair in *Mycobacterium tuberculosis*. What have we learnt from the genome sequence? Mol Microbiol 1998 29 1331 1339 10.1046/j.1365-2958.1998.01038.x 9781872

[B29] Springer B Sander P Sedlacek L Hardt WD Mizrahi V Schär P Böttger EC Lack of mismatch correction facilitates genome evolution in mycobacteria Mol Microbiol 2004 53 1601 1609 10.1111/j.1365-2958.2004.04231.x 15341642

[B30] Hiriyanna KT Ramakrishnan T Deoxyribonucleic acid replication time in *Mycobacterium tuberculosis *H37 Rv Arch Microbiol 1986 144 105 109 10.1007/BF00414718 3087325

[B31] Dos Vultos T Mestre O Tonjum T Gicquel B DNA repair in *Mycobacterium tuberculosis *revisited FEMS 2009 10.1111/j.1574-6976.2009.00170.x 19385996

[B32] Demple B Harrison L Repair of oxidative damage to DNA: enzymology and biology Annu Rev Biochem 1994 63 915 948 10.1146/annurev.bi.63.070194.004411 7979257

[B33] Neeley WL Essigmann JM Mechanisms of formation, genotoxicity, and mutation of guanine oxidation products Chem Res Toxicol 2006 19 491 505 10.1021/tx0600043 16608160

[B34] David SS O'Shea VL Kundu S Base-excision repair of oxidative DNA damage Nature 2007 447 941 950 10.1038/nature05978 17581577 PMC2896554

[B35] Snapper SB Melton RE Mustafa S Kieser T Jacobs WR Jr Isolation and characterization of efficient plasmid transformation mutants of *Mycobacterium smegmatis* Mol Microbiol 1990 4 1911 1919 10.1111/j.1365-2958.1990.tb02040.x 2082148

[B36] Sambrook J Fitsch EF Maniatis T Molecular Cloning: A Laboratory Manual 1989 Cold Spring Harbor, Cold Spring Harbor Press

[B37] Pelicic V Reyrat JM Gicquel B Generation of unmarked directed mutations in mycobacteria, using sucrose counter-selectable suicide vectors Mol Microbiol 1996 20 919 925 10.1111/j.1365-2958.1996.tb02533.x 8809745

[B38] de Mendonca-Lima L Picardeau M Raynaud C Rauzier J de la salmoniere YO Barker L Bigi F Cataldi A Gicquel B Reyrat JM Erp, an extracellular protein family specific to mycobacteria Microbiology 2001 147 2315 2320 11496008 10.1099/00221287-147-8-2315

[B39] Vultos TD Mederle I Abadie V Pimentel M Moniz-Pereira J Gicquel B Reyrat JM Winter N Modification of the mycobacteriophage Ms6 attP core allows the integration of multiple vectors into different tRNAala T-loops in slow- and fast-growing mycobacteria BMC Mol Biol 2006 7 47 10.1186/1471-2199-7-47 17173678 PMC1762012

